# Microfluidic Production of Autofluorescent BSA Hydrogel Microspheres and Their Sequential Trapping for Fluorescence-Based On-Chip Permanganate Sensing

**DOI:** 10.3390/s20205886

**Published:** 2020-10-17

**Authors:** Linbo Liu, Guangming Li, Nan Xiang, Xing Huang, Kota Shiba

**Affiliations:** 1 John A. Paulson School of Engineering and Applied Sciences, Harvard University, Cambridge, MA 02138, USA; linboliu@g.harvard.edu (L.L.); xinghuang@g.harvard.edu (X.H.); 2 Jiangsu Key Laboratory for Design and Manufacture of Micro-Nano Biomedical Instruments, School of Mechanical Engineering, Southeast University, Nanjing 211189, China; nan.xiang@seu.edu.cn; 3 State Key Laboratory of Rare Earth Resource Utilization, Changchun Institute of Applied Chemistry, Changchun 130022, China; 4 State Key Laboratory of Rare Earth Resource Utilization, University of Science and Technology of China, Hefei 230026, China; 5 Institute of Process Equipment, College of Energy Engineering, Zhejiang University, Hangzhou 310027, China; 6 Center for Functional Sensor & Actuator (CFSN), National Institute for Materials Science (NIMS), 1-1 Namiki, Tsukuba, Ibaraki 305-0044, Japan

**Keywords:** droplet-based microfluidics, hydrodynamic trapping, BSA microspheres, autofluorescence, on-chip sensing

## Abstract

Microfabrication technologies have extensively advanced over the past decades, realizing a variety of well-designed compact devices for material synthesis, separation, analysis, monitoring, sensing, and so on. The performance of such devices has been undoubtedly improved, while it is still challenging to build up a platform by rationally combining multiple processes toward practical demands which become more diverse and complicated. Here, we present a simple and effective microfluidic system to produce and immobilize a well-defined functional material for on-chip permanganate (MnO_4_^−^) sensing. A droplet-based microfluidic approach that can continuously produce monodispersed droplets in a water-in-oil system is employed to prepare highly uniform microspheres (average size: 102 μm, coefficient of variation: 3.7%) composed of bovine serum albumin (BSA) hydrogel with autofluorescence properties in the presence of glutaraldehyde (GA). Each BSA hydrogel microsphere is subsequently immobilized in a microchannel with a hydrodynamic trapping structure to serve as an independent fluorescence unit. Various anions such as Cl^−^, NO_3_^−^, PO_4_^3−^, Br^−^, BrO_3_^−^, ClO_4_^−^, SCN^−^, HCO_3_^−^, and MnO_4_^−^ are individually flowed into the microchannel, resulting in significant fluorescence quenching only in the case of MnO_4_^−^. Linear correlation is confirmed at an MnO_4_^−^ concentration from 20 to 80 μM, and a limit of detection is estimated to be 1.7 μM. Furthermore, we demonstrate the simultaneous immobilization of two kinds of different microspheres in parallel microchannels, pure BSA hydrogel microspheres and BSA hydrogel microspheres containing rhodamine B molecules, making it possible to acquire two fluorescence signals (green and yellow). The present microfluidics-based combined approach will be useful to record a fingerprint of complicated samples for sensing/identification purposes by flexibly designing the size and composition of the BSA hydrogel microspheres, immobilizing them in a desired manner and obtaining a specific pattern.

## 1. Introduction

The ability to perform fluorescence sensing in a small-scale system such as a microfluidic chip has attracted much attention because the amount of reagents required for this purpose is tiny, making it inexpensive, less hazardous, and environmentally friendly [1,2,3]. Importantly, such a small analyte volume is also helpful for reducing background signals to achieve a high signal-to-noise ratio [4]. However, it is challenging to integrate a minute amount of conventional fluorescent reagents (e.g., dyes or quantum dots) into a microchannel in a consistent manner, precisely controlling the amount and localization, and stably immobilizing them in the microchannel for consistent device operation [5]. Generally, the immobilization of dyes or quantum dots in a microchannel is technically challenging because their size is much smaller than the channel size of a typical microfluidic device made of polydimethylsiloxane (PDMS) [6]. Thus, a practical approach to overcoming such size difference needs to be adopted. According to previous works, some forces including electric, magnetic, acoustic, and light fields have been utilized to control the motion of particles for lab-on-a-chip applications [7,8,9,10]. Furthermore, hydrodynamic trapping is known as an emerging technique and considered to be a more versatile and promising approach for integrating microscale particles in a microfluidic device. It enables placing the microparticles one by one into an arbitrary location simply on the basis of flow resistance [11]. Until now, this method has been widely used to immobilize droplets or cells, meaning that it would be beneficial to a variety of applications [12,13,14,15,16,17,18,19,20,21,22]. Taking this into account, it is conceivable that, in combination with the hydrodynamic trapping technique, developing new microscale fluorescent materials or microscale carriers for encapsulating conventional fluorescent reagents should be a key to addressing the problem of the dimensional gap.

Droplet-based microfluidics has been known for its unique ability to produce monodispersed droplets with precisely controlled size in a reproducible manner [23,24,25,26,27]. For materials engineering, each generated droplet can serve as an independent reactor and provide a reduced reaction volume compared to bulk experiments to realize fast and efficient mixing. Various materials including organic and inorganic compounds have been prepared in the droplets by dissolving precursor species into the dispersed phase in advance [28,29,30,31,32,33,34]. Among them, hydrogels have been paid great attention to in three-dimensional (3D) cell cultures and analyses because of their biocompatibility and remarkable affinity for water [35,36]. Hydrogels with an open network structure can absorb a large amount of water, making them more suitable for promoting mixing and reaction [37,38,39]. Recently, a hydrogel prepared from bovine serum albumin (BSA) has attracted widespread attention because of its low cost, facile preparation, and excellent accessibility of the ligand binding [40,41,42]. It was found that BSA hydrogel microspheres prepared through the spray-drying of glutaraldehyde (GA) cross-linked BSA showed strong autofluorescence [43]. Additionally, the same hydrogel microspheres were used to study enzymatic degradation with the assistance of the autofluorescence property [44]. Although there were some studies which focused on applications of the autofluorescent BSA hydrogel microspheres as described, no work achieved the preparation of uniform-sized autofluorescent BSA hydrogel microspheres using droplet-based microfluidics. Droplets generated by the microfluidic technique are regarded as an ideal reactor for preparing highly uniform autofluorescent BSA hydrogel microspheres.

In this paper, we introduce a facile microfluidic approach to preparing monodispersed autofluorescent BSA hydrogel microspheres and immobilize them into a microchannel for fluorescence-based on-chip sensing of MnO_4_^−^. The highly uniform autofluorescent BSA hydrogel microspheres are simply obtained via the cross-linking reaction between BSA and glutaraldehyde (GA) confined in a large number of independent droplets that are generated from a droplet-based microfluidic device. Taking advantage of the hydrodynamic trapping, we successfully place the autofluorescent BSA hydrogel microspheres into the designated locations one by one in parallel microfluidic channels. We then show that the autofluorescent BSA hydrogel microspheres in the microchannel serve as effective fluorescence sensors for sensitive and selective detection of MnO_4_^−^ with a good linear range from 20 to 80 µM. Potassium permanganate (KMnO_4_) is a well-known strong oxidant which widely serves as a disinfectant in our daily life or is used in the treatment of pollutants in water [45,46]. However, excess MnO_4_^−^ in drinking or environmental water can lead to respiratory infections and skin irritation [47,48,49]. Therefore, the content of residual MnO_4_^−^ in treated water should be strictly monitored. Finally, we demonstrate the immobilization of autofluorescent BSA hydrogel microspheres loaded with/without rhodamine B molecules in parallel microchannels, realizing the simultaneous acquisition of two fluorescence signals (yellow and green).

## 2. Materials and Methods

### 2.1. Chemicals

BSA, rhodamine B, sodium chloride (NaCl), sodium nitrate (NaNO_3_), trisodium phosphate (Na_3_PO_4_), sodium bromide (NaBr), sodium perchlorate (NaClO_4_), sodium thiocyanate (NaSCN), and sodium bicarbonate (NaHCO_3_) were purchased from Sigma-Aldrich Corporation. Propylene glycol methyl ether acetate (PGMEA) and SU-8 3050 were purchased from MicroChem Corporation. PDMS (base elastomer and curing agent) was purchased from Dow Corning Corporation. GA solution (50 wt.%) was purchased from VWR Corporation. KMnO_4_ was purchased from EMD Millipore Corporation. Fluorocarbon oil (HFE7500) was bought from the 3M Company.

### 2.2. Microfluidic Device Fabrication

Briefly, negative photoresist SU-8 3050 was spun onto a cleaned 3 inch single-side-polished silicon wafer through a spin-coating procedure to form a 50 µm thick layer. After baking at 95 °C for 15 min, the photoresist layer was covered with a printed photomask and then exposed to ultraviolet (UV) light. Subsequently, the mask was removed from the wafer, and the wafer was baked again at 95 °C. The master mold with microchannel structures was obtained after dissolving the uncured resist with propylene glycol monomethyl ether acetate (PGMEA). After that, the master mold was transferred onto a hot plate set at 200 °C for 5 min for stabilization. Then, a micro-molding procedure was adopted to replicate the microchannel from the master mold. The mixture (weight ratio of 10:1) of PDMS liquid with base and curing agent was poured on the SU-8 master mold followed by degassing. The cured PDMS was peeled off from the master mold after curing at 65 °C for a couple of hours. After making through-holes as inlet and outlet, the PDMS block was irreversibly bonded with a glass slide via plasma treatment. The microfluidic chip fabrication was completed after enhancing the bonding strength at 65 °C for several hours.

### 2.3. Synthesis of Autofluorescent BSA Hydrogel Microspheres

BSA solution (225 mg/mL) was simply obtained by dissolving BSA powder into deionized (DI) water under ultrasonication. GA solution (5 wt.%) was obtained by diluting commercial GA aqueous solution (50 wt.%) into DI water. The oil phase was HFE7500 with 2% Krytox–PEG–Krytox surfactant. All solutions were loaded into syringes, and then the syringes were connected to the microfluidic chip via needles and polyethylene tubing with an inner diameter of 0.38 mm (Scientific Commodities, Inc. Lake Havasu City, AZ, USA). The flow rates were individually controlled by pumps (PHD 2000 Infusion, Harvard Apparatus, Boston, MA, USA), and the flow was observed using an inverted microscope connected with a high-speed camera. The flow rate of two aqueous phases was set at 500 μL/h, while the flow rate of the oil phase was set at 1000 μL/h. The droplets were collected into HFE7500 covered by a small amount of silicone oil for preventing HFE7500 from evaporating and then left overnight at room temperature. The final product was directly used in the next step without any further treatment.

### 2.4. Immobilization of BSA Hydrogel Microspheres into Microchannels

The autofluorescent BSA hydrogel microspheres were injected into a microfluidic chip with the hydrodynamic trapping structure. Most of the autofluorescent BSA hydrogel microspheres were caught by the traps located in the microfluidic chip. Free autofluorescent BSA hydrogel microspheres remaining in the microchannels were washed out by injecting pure HFE7500. Then, the air was carefully flowed through the microchannel to remove HFE7500, and the microfluidic chip was dried overnight at room temperature.

### 2.5. Fluorescence-Based Anion Sensing

The present fluorescence-based various anion sensing was performed using the microfluidic chip after trapping the autofluorescent BSA hydrogel microspheres. In a typical assay, MnO_4_^−^ solution with different concentrations such as 0, 20, 30, 40, 50, 60, and 80 μM (pH = 3) was injected into the microfluidic chip with a syringe pump. The flow rate was set at 4.8 mL/h, and the injection time was 10 min. Fluorescence microscope images were taken to study the fluorescence changes of the autofluorescent BSA hydrogel microspheres under the same conditions. The selectivity for MnO_4_^−^ was confirmed by injecting other anion solutions such as Cl^−^, NO_3_^−^, PO_4_^3−^, Br^−^, BrO_3_^−^, ClO_4_^−^, SCN^−^, and HCO_3_^−^. The first autofluorescent BSA microspheres trapped in all the eight parallel channels were focused and used for all the calculations. To minimize the measurement error, the laser was illuminated to the autofluorescent BSA hydrogel microsphere only at the specified time point. 

## 3. Results and Discussion

**Scheme 1:**
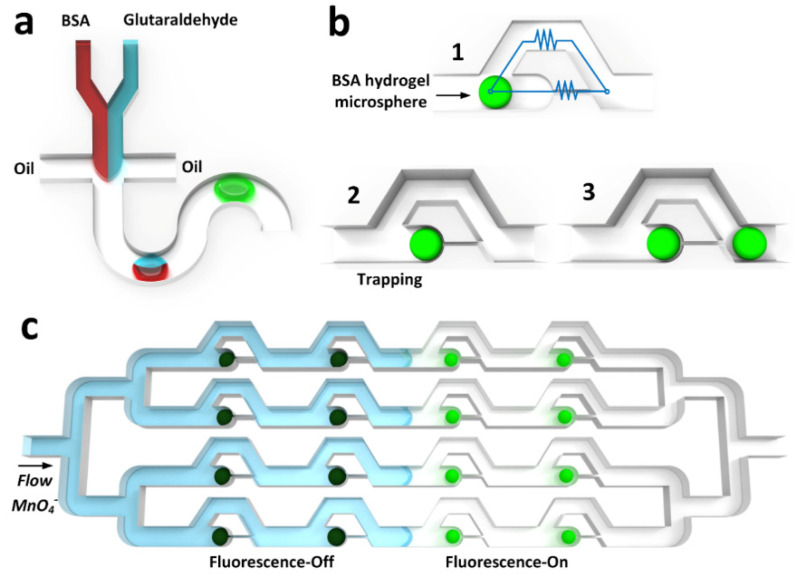
(**a**) The preparation of the autofluorescent bovine serum albumin (BSA) hydrogel microspheres via droplet-based microfluidics; (**b**) the immobilization of the autofluorescent BSA hydrogel microspheres into parallel microchannels via hydrodynamic trapping; (**c**) fluorescence-based on-chip sensing of MnO_4_^−^.

Scheme 1 illustrates the design rationale. The synthesis of the autofluorescent BSA hydrogel microspheres was performed with a well-known flow-focusing microfluidic device (Scheme 1a) [50]. The microfluidic device contained three inlets and a long serpentine flowing channel. The serpentine flowing channel here was used for the rapid mixing of two aqueous phases (BSA and GA) in the droplets. The two aqueous phases and oil phase were injected through the three inlets separately. Droplets were obtained by shearing of oil phase to aqueous phase. Scheme 1b shows the present design for immobilizing the autofluorescent BSA hydrogel microspheres into the microchannel via hydrodynamic trapping. Since the opening of a capture port is parallel to the flow direction of the BSA hydrogel microsphere and the flow resistance of the main channel is smaller than the side channel, the BSA hydrogel microsphere can enter the capture port and be captured. After one BSA hydrogel microsphere plugs the entrance of a narrow channel just behind the port, the following BSA hydrogel microspheres can flow into the side channel and push the former BSA hydrogel microsphere to stay in the capture port more tightly. After drying, the microsphere-immobilized chip can serve as an on-chip on/off fluorescence sensor for MnO_4_^−^ (Scheme 1c).

**Figure 1:**
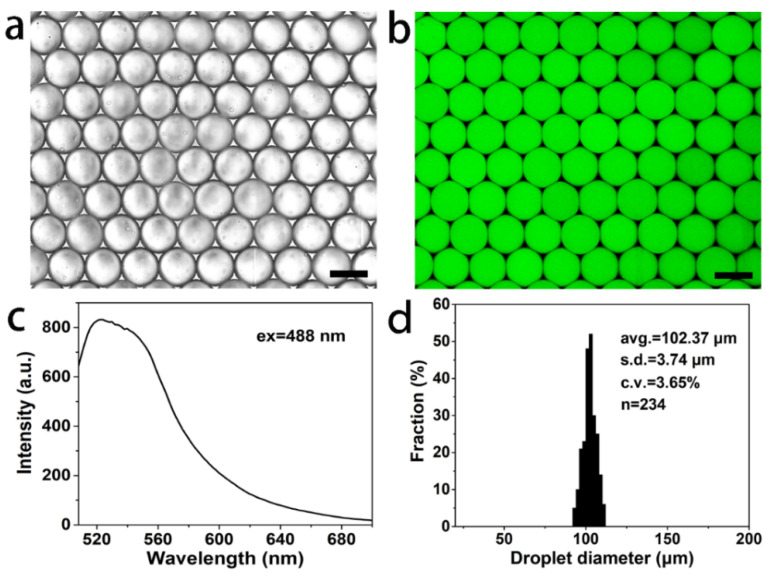
(**a**) Optical microscope image of the autofluorescent BSA hydrogel microspheres; (**b**) fluorescence microscope image of the autofluorescent BSA hydrogel microspheres (excited at 488 nm). All scale bars are 100 µm; (**c**) photoluminescence emission spectra of the autofluorescent BSA hydrogel microspheres; (**d**) size distribution of the autofluorescent BSA hydrogel microspheres.

Droplets can be continuously prepared by shearing of oil phase to aqueous phase (Video S1, Supplementary Materials). The interface between the two aqueous phases disappeared quickly, meaning that the BSA and GA solutions were mixed well in the serpentine channel (Figure S1, Supplementary Materials). Each BSA hydrogel microsphere was produced by the cross-linking reaction between BSA and GA in a discrete droplet. The optical microscope images were taken to examine the shape and size of the BSA hydrogel microspheres. Figure 1a shows that the autofluorescent BSA hydrogel microspheres were spherical and monodispersed. These autofluorescent BSA hydrogel microspheres showed clear green emission under 488 nm laser irradiation (Figure 1b). The emission spectrum of the BSA hydrogel microspheres showed a broad green emission peak (at around 510–560 nm) excited by 488 nm (Figure 1c), which was consistent with the fluorescence microscope image. To confirm the size distribution, the sizes of the autofluorescent BSA hydrogel microspheres were measured by ImageJ (Figure 1d). On the basis of 234 microspheres, the average diameter was estimated to be 102 μm with a coefficient of variation of 3.7%. The size distribution of the present autofluorescent BSA hydrogel microspheres was narrower than that reported in the previous study [43]. It is worth noting that the size variation in the autofluorescent BSA hydrogel microspheres could cause deviations in quantification for fluorescence-based sensing.

**Figure 2:**
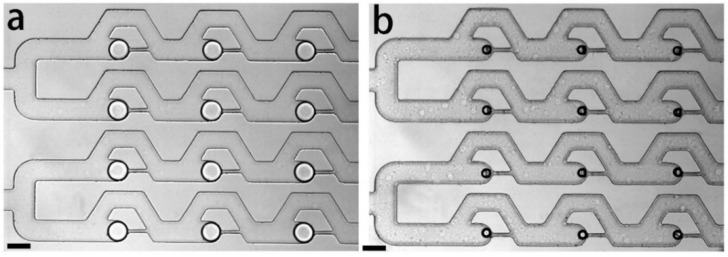
Optical microscope images of the autofluorescent BSA hydrogel microspheres immobilized in microchannels through hydrodynamic trapping (**a**) before and (**b**) after drying. All scale bars are 100 µm.

The as-prepared autofluorescent BSA hydrogel microspheres were injected into the parallel microchannels using a syringe (Videos S2 and S3, Supplementary Materials). It should be noted here that the efficiency of the loading achieved 100%. Every BSA hydrogel microsphere was caught by a trap because their sizes were well matched in the present study for single-microsphere capture (Figure 2a). Before sensing MnO_4_^−^ in water, HFE7500 was removed by carefully flowing air into the microchannels first and then dried at room temperature overnight (Videos S4 and S5, Supplementary Materials). As shown in Figure 2b, although the size of the dried autofluorescent BSA microspheres was much smaller than that of the original autofluorescent BSA microspheres, they remained in the traps even after the drying process. Compared to the original autofluorescent BSA hydrogel microspheres, they still showed strong fluorescence emission after drying (Figure S2, Supplementary Materials).

**Figure 3:**
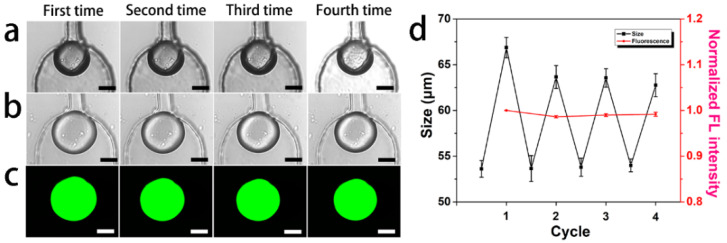
(**a**) Optical microscope images of the dried autofluorescent BSA hydrogel microspheres, (**b**) after injecting water in a trap, and (**c**) the corresponding fluorescence microscope images (all scale bars are 25 µm); (**d**) cycle number-dependent change of size (black line) and fluorescence intensity (red line) of the autofluorescent BSA hydrogel microspheres. The error bars represent the standard deviation of three measurements. The drying–swelling cycle was repeated four times.

The water absorption behavior of the autofluorescent BSA hydrogel microsphere was studied under optical microscope observation. The size of the dried BSA hydrogel microsphere obviously increased after infusing water into the microchannel (Figure 3a,b), and good reversibility was confirmed through multiple drying–swelling cycles (Figure 3d). Notably, a rapid increase in size was clearly observed during water absorption (Video S6, Supplementary Materials). After swelling, the size of the BSA hydrogel microsphere only recovered up to around 60% of the initial value (102 μm) probably because the original one contained an excess amount of water in its whole structure. No significant size variation was seen after the first drying–swelling cycle. Considering that the fluorescence intensity of the autofluorescent BSA hydrogel microsphere was also constant before and after the cycles (Figure 3c,d), the present microspheres reached a stable state. It should be noted that the position of the dried BSA hydrogel microsphere did not change during repeated water absorption, indicating that it can be continuously monitored in the traps.

**Figure 4:**
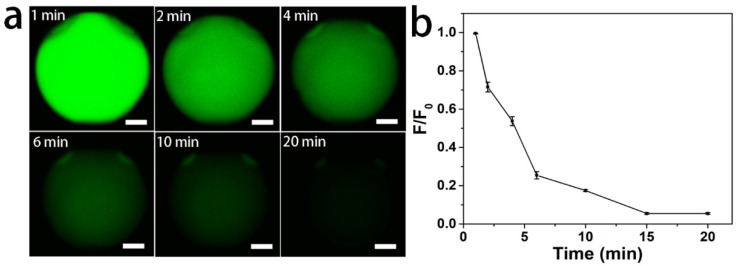
(**a**) Fluorescence microscope images of autofluorescent BSA sphere at different injection times and (**b**) the corresponding F/F_0_ curve of the autofluorescent BSA hydrogel microspheres in the presence of MnO_4_^−^ (80 µM). F and F_0_ are the fluorescence intensities of the autofluorescent BSA hydrogel microspheres excited at 488 nm in the presence and absence of MnO_4_^−^, respectively. All scale bars are 10 µm. The error bars represent the standard deviation of three measurements.

For the present sensing application, we first tested if the MnO_4_^−^ could directly quench the autofluorescence of the as-prepared BSA hydrogel microspheres. As shown in Figure S3 (Supplementary Materials), MnO_4_^−^ solution had almost no fluorescence-quenching effect on the BSA hydrogel microspheres under neutral conditions. However, MnO_4_^−^ solution could effectively quench the fluorescence of the BSA hydrogel microspheres in acidic conditions (pH = 3). The reason for this phenomenon could be attributed to the enhancement of the MnO_4_^−^ oxidizing ability in the presence of acid. Then, we studied the effect of injection time on fluorescence quenching of the BSA hydrogel microspheres in the presence of MnO_4_^−^ (80 µM). As shown in Figure 4, the fluorescence intensity decreased rapidly within 6 min, and then started slowing down. The fluorescence intensity changed only a little between 10 and 20 min, suggesting that 10 min would be the optimal fluorescence test time for sensing MnO_4_^−^. 

**Figure 5:**
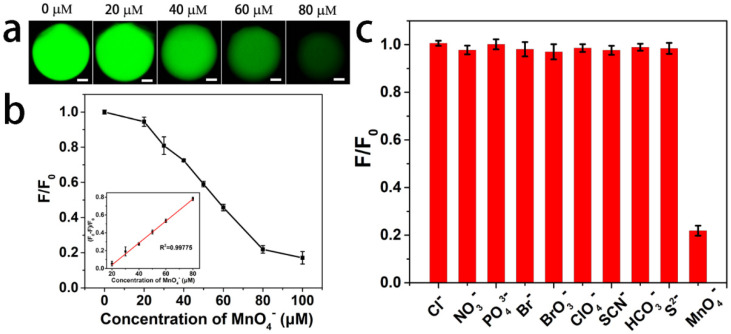
(**a**) Fluorescence microscope images of the autofluorescent BSA hydrogel microspheres in the presence of MnO_4_^−^ at different concentrations (pH = 3) (All the scale bars are 10 µm); (**b**) the concentration titration curve between F/F_0_ and the concentration of MnO_4_^−^ (inset figure: linear relationship between (F_0_ − F)/F_0_ and the concentration of MnO_4_^−^); (**c**) relative fluorescence intensity of the autofluorescent BSA hydrogel microspheres after being exposed to various analytes (the concentration of all the anions was set at 80 µM). The error bars represent the standard deviation of three measurements.

Subsequently, the fluorescence intensity changes of the BSA hydrogel microspheres to MnO_4_^−^ concentrations (pH = 3) were studied. Figure 5a shows that the fluorescence intensity of autofluorescent BSA hydrogel microspheres gradually decreased as the concentration of MnO_4_^−^ increased from 20 to 80 µM. The complete concentration titration curve shows that obvious quenching occurred at 20 µM and stops at 80 µM as shown in the Figure 5b. The quenching efficiency (F_0_ − F)/F_0_ showed a good linearity (*R* = 0.99775) versus the concentration of MnO_4_^−^ in the range of 20–80 µM (inset of Figure 5b). The limit of detection according to the 3σ/slope was estimated to be 1.7 µM. The autofluorescent BSA hydrogel microspheres trapped at the same position in each parallel channel could serve as a set of control experiments, which were used to reduce the error caused by repeated operations. Importantly, the realization of each autofluorescent BSA hydrogel microsphere as an independent detection unit makes the present approach more efficient, low-cost, and even environmentally friendly. The selectivity of the autofluorescent BSA hydrogel microspheres to MnO_4_^−^ was also studied. Compared to some possible coexisting species, only MnO_4_^−^ greatly quenched the fluorescence of the autofluorescent BSA hydrogel microspheres in the same conditions, indicating that the as-prepared BSA hydrogel microspheres have good selectivity to MnO_4_^−^ (Figure 5c). Considering that ClO_4_^−^ and BrO_3_^−^ also have strong oxidizing ability under acidic conditions but did not quench fluorescence of the autofluorescent BSA hydrogel microsphere, we hypothesize that the quenching-based detection process involves the formation of the MnO_2_ particles or Mn-based precipitates seen in Figure S4 (Supplementary Materials). The Mn-based species prevented the autofluorescent BSA microspheres from being excited and/or emitting fluorescence. Detailed studies on the subsequent applications and reaction mechanisms of these sensors are now in progress and will be reported elsewhere.

**Figure 6:**
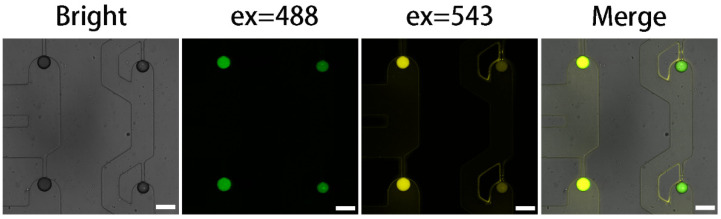
Optical and fluorescence microscope images of the autofluorescent BSA hydrogel microspheres (**left**) with and (**right**) without rhodamine B in parallel microchannels. All scale bars are 100 µm.

Finally, we demonstrated the immobilization of two different BSA hydrogel microspheres with/without guest species (rhodamine B) into parallel microchannels. The synthesis process of the dyed BSA hydrogel microspheres was similar to that of the pure BSA hydrogel microspheres, except that rhodamine B was added to the initial BSA solution. The size of the dyed BSA hydrogel microspheres was approximately 1.5-fold larger than pure BSA hydrogel microspheres. As shown in Figure S5 (Supplementary Materials), the principle of the present design was based on the size matching of hydrogel microspheres and traps. Larger hydrogel microspheres were injected first and caught in the larger traps. Afterward, smaller hydrogel microspheres were injected and caught in the smaller traps. Figure 6 shows that the dried dyed BSA hydrogel microspheres and dried pure BSA hydrogel microspheres stayed in the correct traps. Both types of microspheres emitted green and yellow fluorescence when they were excited by 488 and 543 nm lasers, respectively. Interestingly, single yellow and green signals were obtained in different channels from the merged image, indicating that rhodamine B could exist homogeneously dispersed in the dried BSA hydrogel microspheres and maintain its original fluorescence property. The result indicates that the BSA hydrogel microsphere is a suitable matrix for small dye molecules, leading to various microfluidic applications such as visual detection and identification.

## 4. Conclusions

We synthesized highly uniform autofluorescent BSA hydrogel microspheres via a droplet microfluidic technique for the first time. The autofluorescent BSA hydrogel microspheres were immobilized into parallel microchannels via a hydrodynamic trapping technique, resulting in multiple independent microsphere arrays. The entire immobilization process was easily controlled as a function of flow resistance. The autofluorescent BSA microspheres remained in the preset detection position even after multiple drying–swelling cycles with air and water injection. Each microsphere served as a fluorescence sensing unit for MnO_4_^−^ with high sensitivity and selectivity. Our entire design has made significant progress compared to previous approaches in several aspects. First, since the opening of a capture port is parallel to the flow direction and each particle must pass through the capture structure, nearly 100% capture rate is achieved. Second, multiple flow channels are arranged in parallel to achieve high throughput and make the detection more efficient. Our structure is also simpler and more compact. Third, multiple detection results under the same conditions can be obtained from a single operation, making the detection results more reliable and accurate. Lastly, we also realized simultaneous acquisition of two fluorescence signals (yellow and green) by trapping the autofluorescent BSA hydrogel microspheres loaded with/without rhodamine B molecules in parallel microchannels. Two kinds of microspheres with different fluorescence emissions in parallel microchannels have potential applications in the construction of sensors as a function of fluorescence intensity ratio or simultaneous detection of multiple substances. Immobilizing a wider variety of microspheres in a microchannel will display a unique pattern which is a fingerprint of analytes, leading to the detection/identification of complicated samples such as body fluids.

## Supplementary Materials

The following are available online at https://www.mdpi.com/1424-8220/20/20/5886/s1: **Figure S1**. Optical microscope image of the present droplet generation device; **Figure S2**. (a) Optical and fluorescence microscope images of the autofluorescent BSA hydrogel microsphere and (b) dried autofluorescent BSA hydrogel microspheres immobilized in the microchannel. All scale bars are 25 µm; **Figure S3**. pH-dependent fluorescence property of the autofluorescent BSA hydrogel microspheres in the presence of MnO_4_^−^ (80 µM); **Figure S4**. Optical microscope images of the autofluorescent BSA hydrogel microspheres in the presence of MnO_4_^−^ at different concentrations (pH = 3). All scale bars are 10 µm; **Figure S5**. Microfluidic chip design for immobilizing the autofluorescent BSA hydrogel microspheres loaded with rhodamine B dye (bigger traps) and pure BSA hydrogel microspheres (smaller traps); **Video S1**. Droplet generation; **Video S2**. Trapping process; **Video S3**. Overview after trapping; **Video S4**. Drying process; **Video S5**. Overview after drying; **Video S6**. Water absorption.
