# Supplementary material for: Microfluidic Production of Autofluorescent BSA Hydrogel Microspheres and Their Sequential Trapping for Fluorescence-Based On-Chip Permanganate Sensing
Source: Sensors (Basel). 2020 Oct 17;20(20):5886. doi: 10.3390/s20205886 (PMC7594029; doi:10.3390/s20205886)
Supplement: Supplementary file 1 [file sensors-20-05886-s001.zip › supplementary materials.pdf]

Supporting Information

Microfluidic Production of Autofluorescent BSA Hydrogel Microspheres and Their Sequential Trapping for Fluorescence-Based On-Chip Permanganate Sensing

Linbo Liu [1,2], Guangming Li [1,3,4,*], Nan Xiang [2], Xing Huang [1,5] and Kota Shiba [1,6,*]

1. John A. Paulson School of Engineering and Applied Sciences, Harvard University, Cambridge, Massachusetts 02138, USA
2. School of Mechanical Engineering, and Jiangsu Key Laboratory for Design and Manufacture of Micro-Nano Biomedical Instruments, Southeast University, Nanjing 211189, China
3. State Key Laboratory of Rare Earth Resource Utilization, Changchun Institute of Applied Chemistry, Changchun 130022, China
4. University of Science and Technology of China, Hefei 230026, China
5. Institute of Process Equipment, College of Energy Engineering, Zhejiang University, Hangzhou, 310027, China
6. Center for Functional Sensor & Actuator (CFSN), National Institute for Materials Science (NIMS), 1-1 Namiki, Tsukuba, Ibaraki 305-0044, Japan

To whom correspondence should be addressed:

guangmingli2020@163.com, kshiba@seas.harvard.edu, SHIBA.Kota@nims.go.jp

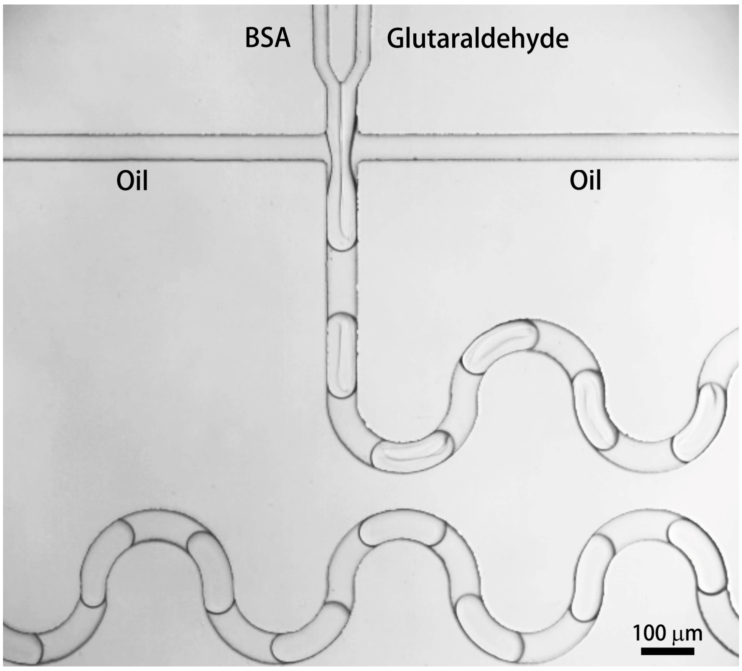

Figure S1. Optical microscope image of the present droplet generation device.

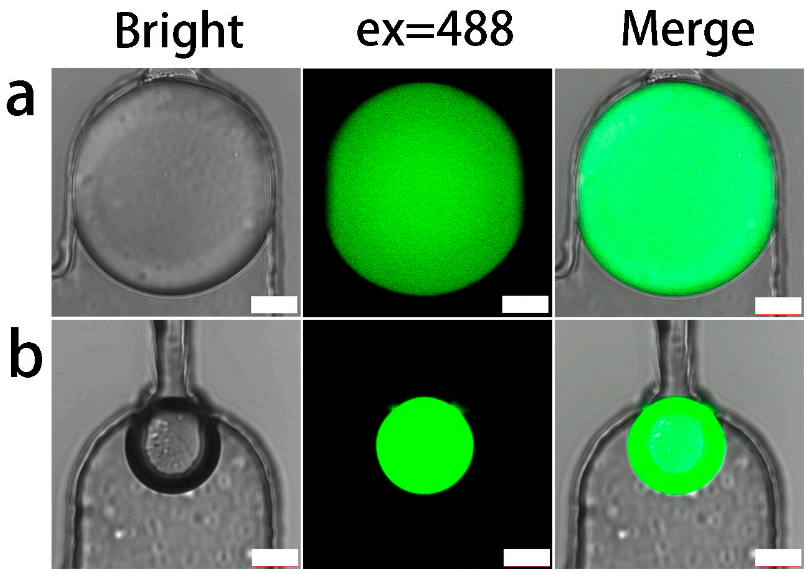

Figure S2. (a) Optical and fluorescence microscope images of the autofluorescent BSA hydrogel microsphere and (b) dried autofluorescent BSA hydrogel microsphere immobilized in the microchannel. All the scale bars are 25 μm.

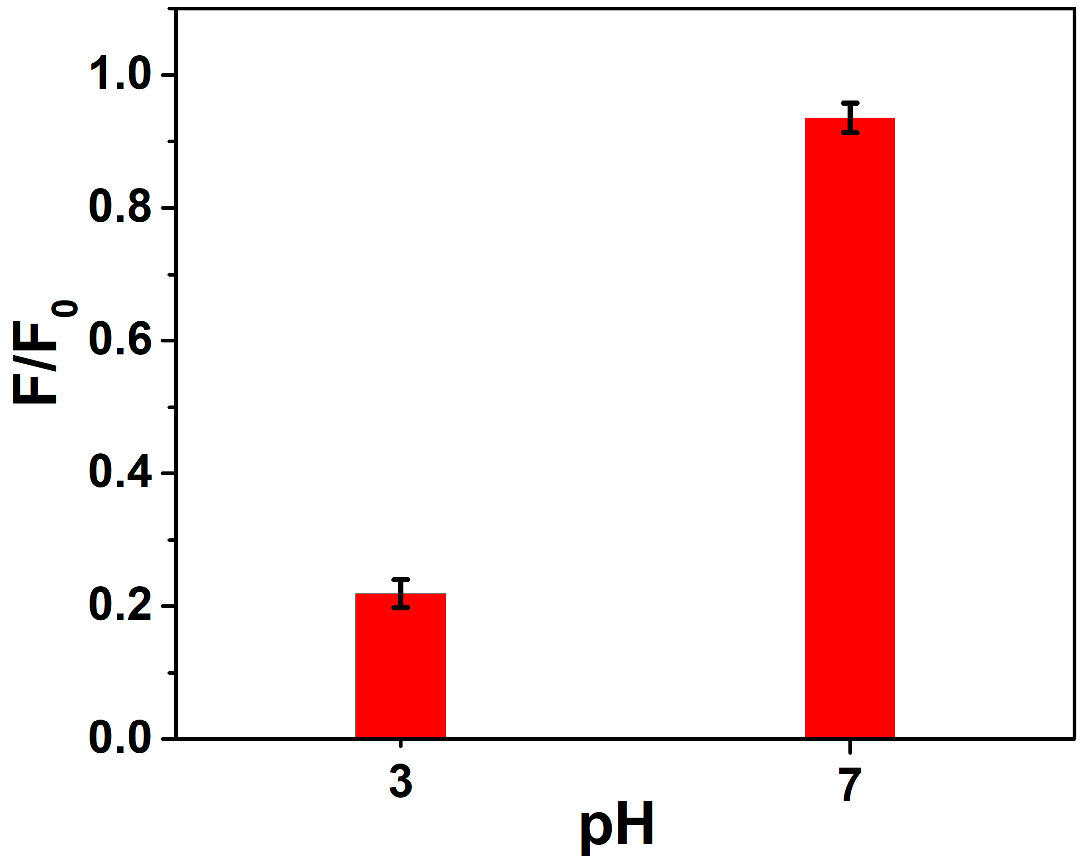

Figure S3. pH-dependent fluorescence property of the autofluorescent BSA hydrogel microspheres in the presence of MnO_4^- (80 μM).

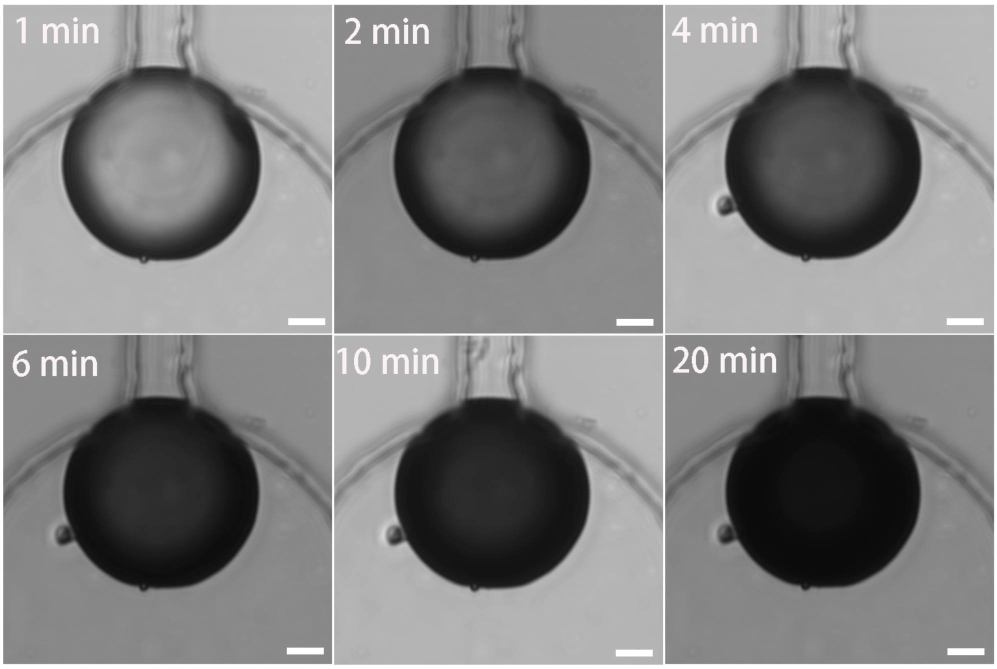

Figure S4. Optical microscope images of the autofluorescent BSA hydrogel microspheres in the presence of MnO_4^- at different concentrations (pH=3). All the scale bars are 10 μm.

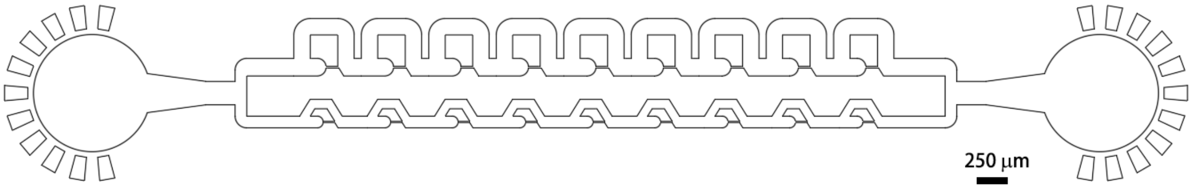

Figure S5. Microfluidic chip design for immobilizing the autofluorescent BSA hydrogel microspheres loaded with rhodamine B dye (bigger traps) and pure BSA hydrogel microspheres (smaller traps).
